# Digital Fatigue and Cognitive Load in E‐Learning: Evidence From Nursing Students in Jordan

**DOI:** 10.1002/nop2.70795

**Published:** 2026-09-02

**Authors:** Shaban Sinnokrot, Randa Khirfan, Ahmad I. Miqdadi, Yousef Abu‐Wardeh, Ala'a Ayman Al‐yyan, Qusai Abdulrahman AbuQamar, Nawras Fashafsheh

**Affiliations:** ^1^ Faculty of Nursing Zarqa University (ZU) Zarqa Jordan; ^2^ Health Management and Hospital Administration, Faculty of Nursing Middle East University Amman Jordan; ^3^ Department of Community & Mental Health Nursing, Faculty of Nursing Zarqa University Zarqa Jordan; ^4^ Department of Clinical Nursing, Faculty of Nursing Zarqa University Zarqa Jordan; ^5^ Faculty of Nursing Arab American University Palestine Jenin Palestine; ^6^ Department of Nursing, Faculty of Health Sciences Istanbul Gelisim University Istanbul Turkey

## Abstract

**Aim:**

To assess digital fatigue and multidimensional cognitive load, and to examine their associated factors among nursing students engaged in online learning in Jordanian universities.

**Design:**

A cross‐sectional study.

**Methods:**

An Internet‐based questionnaire was distributed to undergraduate nursing students from governmental and private universities in Jordan between March and April 2025. Digital fatigue was measured using the Zoom Exhaustion and Fatigue scale, and cognitive load was measured using Leppink's Cognitive Load Scale. Associations were examined using bivariate tests, and a multiple linear regression analysis with bootstrapping was conducted to identify factors independently associated with extrinsic cognitive load.

**Results:**

A total of 537 nursing students participated. Students reported relatively high levels of digital fatigue and cognitive load, with extrinsic cognitive load showing the pronounced ceiling effect. Digital fatigue was independently associated with extrinsic cognitive load after adjusting for demographic and academic characteristics. Academic year, type of university, and daily digital device use were also independently associated with extrinsic cognitive load, while age, sex, grade point average, device type, and Internet quality showed no significant associations.

**Conclusion:**

Digital fatigue was independently associated with extrinsic cognitive load among nursing students, highlighting the role of instructional design in shaping students' cognitive experiences.

**Implications for the Profession and/or Patient Care:**

Nurse educators should prioritise instructional strategies that reduce unnecessary cognitive demands in online learning environments, such as simplified navigation, reduced task‐switching, and structured pacing, to support nursing students' cognitive wellbeing.

**Impact:**

Digital fatigue and cognitive load remain underexamined among nursing students in resource‐limited settings. This study identifies extrinsic cognitive load as a modifiable, instructional‐design‐related factor, offering nurse educators a concrete target for improving online nursing education.

**Reporting Method:**

This study adhered to the Strengthening the Reporting of Observational Studies in Epidemiology checklist for cross‐sectional studies.

**Patient or Public Contribution:**

No patient or public contribution.

## 1 Introduction

Digital technology is transforming higher education all over the world, and online and blended learning methods are the most popular (Khatatbeh et al. 2024). This was especially the case after the COVID‐19 pandemic, when universities had to rapidly switch to online platforms to maintain continuity of education (O'Neill 2024; Almaiah et al. 2020). Although these methods enabled greater accessibility and flexibility, they also posed challenges for students in terms of cognitive productivity, learning efficiency, and continuous connection to digital learning environments (Yosep et al. 2023).

Digital fatigue (DF) and cognitive load (CL) are two important constructs that influence students' learning experience online (Çelik Durmuş et al. 2022; Hadie et al. 2021). DF is physical, cognitive and psychological fatigue related to digital devices, screens and virtual learning environments and is commonly referred to as ‘Zoom fatigue’ or ‘screen fatigue’ (Haanes et al. 2024). It is associated with a lack of attention, visual discomfort, lack of motivation, mental fatigue and less learning in education (Kaewpradit et al. 2025; Rayan 2023; Yosep et al. 2023). CL is the mental effort required to process, organise and retain information during learning activities, which is essential to enhance learning efficiency in digital environments (Huang and Fang 2023; Surbakti et al. 2024).

For nursing education, these challenges are especially relevant because students are expected to integrate theoretical knowledge with clinically oriented reasoning and decision‐making under conditions of uncertainty (Alarabiat 2024). Unlike many non‐clinical academic disciplines, nursing education requires learners to apply abstract biomedical concepts to dynamic, high‐stakes patient care situations, a process that imposes substantial cognitive demands (Skulmowski and Xu 2022).

Although DF and CL have been studied in higher education, most studies have studied DF separately or in general university populations (AlOsta et al. 2023; Hadie et al. 2021). This approach might gloss over the unique educational and cognitive burden of nursing students and underestimate the impact of digital learning on students' cognitive ability (Hadie et al. 2021). In addition, there is little evidence on how different CL dimensions (intrinsic, extrinsic and germane load) are related to DF in nursing students (Skulmowski and Xu 2022).

In addition, the evidence is from well‐resourced educational systems and is not necessarily consistent with learning in developing or resource‐poor settings such as Jordan and the Middle East (Almaiah et al. 2020; Wang et al. 2026). Digital infrastructure, access to learning technology and educational support systems may have different impacts on students' online learning and how the findings can be applied in different contexts (O'Neill 2024).

Cognitive Load Theory (CLT) provides a useful theoretical framework for understanding these relationships by distinguishing among intrinsic, extrinsic and germane CL (Sweller et al. 2019; Sepp et al. 2019). Intrinsic load reflects the inherent complexity of learning tasks, germane load represents cognitive effort directed toward schema construction and meaningful learning, whereas extrinsic load reflects unnecessary cognitive effort generated by instructional design and information presentation (Mayer 2021). Because Instructional conditions substantially shape students' cognitive experiences, CLT is particularly relevant for examining how digital learning environments can contribute to fatigue and cognitive burden.

Among the dimensions of CL, extrinsic CL may warrant particular attention in digital learning environments because, unlike intrinsic load, it does not originate from the inherent complexity of the learning material and is therefore more amenable to modification through instructional design (Faudzi et al. 2024; Ouwehand et al. 2025). According to CLT, extrinsic CL arises from unnecessary cognitive demands imposed by instructional design in the way information is presented (Sweller et al. 2019). In online learning environments, factors such as complex digital interfaces, fragmented content delivery, excessive information presentation and inefficient interaction requirements may increase unnecessary mental effort and interfere with learning processes (Skulmowski and Xu 2022). This issue may be especially relevant in nursing education, where students are required to simultaneously integrate theoretical knowledge with clinically oriented reasoning. Consequently, examining extrinsic CL may provide valuable insight into modifiable features of digital learning environments that could reduce unnecessary cognitive burden and potentially alleviate DF while improving students learning experiences (Mayer 2021).

Despite the relevance of CLT for understanding students' cognitive experiences in digital learning, relatively few studies have simultaneously examined DF and multidimensional CL among nursing students, and even fewer studies have explored the role of extrinsic CL as a potentially modifiable source of cognitive burden in online learning environments, particularly in developing contexts such as Jordan (Rahmi et al. 2025; Sepp et al. 2019).

Therefore, guided by CLT, this study aimed to assess DF and multidimensional CL and to examine their associated factors among nursing students engaged in online learning in Jordanian universities.

## 2 Materials and Method

### 2.1 Study Design and Participants

This cross‐sectional study presented an internet‐based questionnaire through Google Forms and shared the survey link through students' email accounts and institutional learning platforms. The study was described in depth in the questionnaire so students could be fully informed before they opted to participate in it. The study was reported according to the Strengthening the Reporting of Observational Studies in Epidemiology (STROBE) guidelines (Supporting Information S1). Data were collected from March to April 2025.

Undergraduate nursing students from five nursing colleges (two governmental and three private) in Jordan were invited to participate. The participating institutions included the University of Jordan, Yarmouk University, Al‐Zaytoonah University of Jordan, Zarqa University, and Applied Science Private University. The study was ethically approved by the Research Ethics Committee, Faculty of Nursing, Zarqa University (74/2024–2025). No separate ethical approval was required from the participating institutions. To facilitate data collection, an official letter of facilitation was obtained from Zarqa University and submitted to the Ministry of Higher Education and Scientific Research, which subsequently communicated with the participating universities to facilitate access to the institutions and data collection. The necessary administrative permissions were obtained from the relevant authorities at each participating institution prior to data collection.

Inclusion criteria were: (1) being enrolled in a nursing program, (2) having at least one online course, and (3) being willing to take part in the study and give informed consent. Exclusion criteria were: (1) students taking drugs that have the potential to affect concentration or cognitive function and (2) students who have not participated in any online course in the last 6 months. Convenience sampling was utilised as it was easy to recruit eligible nursing students from different governmental and private universities and also allowed us to collect data online from different institutions.

The sample size was determined using G*Power 3.1 by multiple linear regression analysis. The calculation was based on an alpha level of 0.05, a medium effect size (*f* ^2^ = 0.15), a desired statistical power of 0.90 and 15 predictors. The 0.90 statistical power was chosen to limit the risk of Type II error and ensure adequate sensitivity to detect significant associations within the multiple linear regression model. Based on these parameters, the minimum required sample size was 171 participants. This level of power is recommended in behavioural and health sciences studies where more precision is needed than the 0.80 standard (Cohen 1988).

**FIGURE 1:**
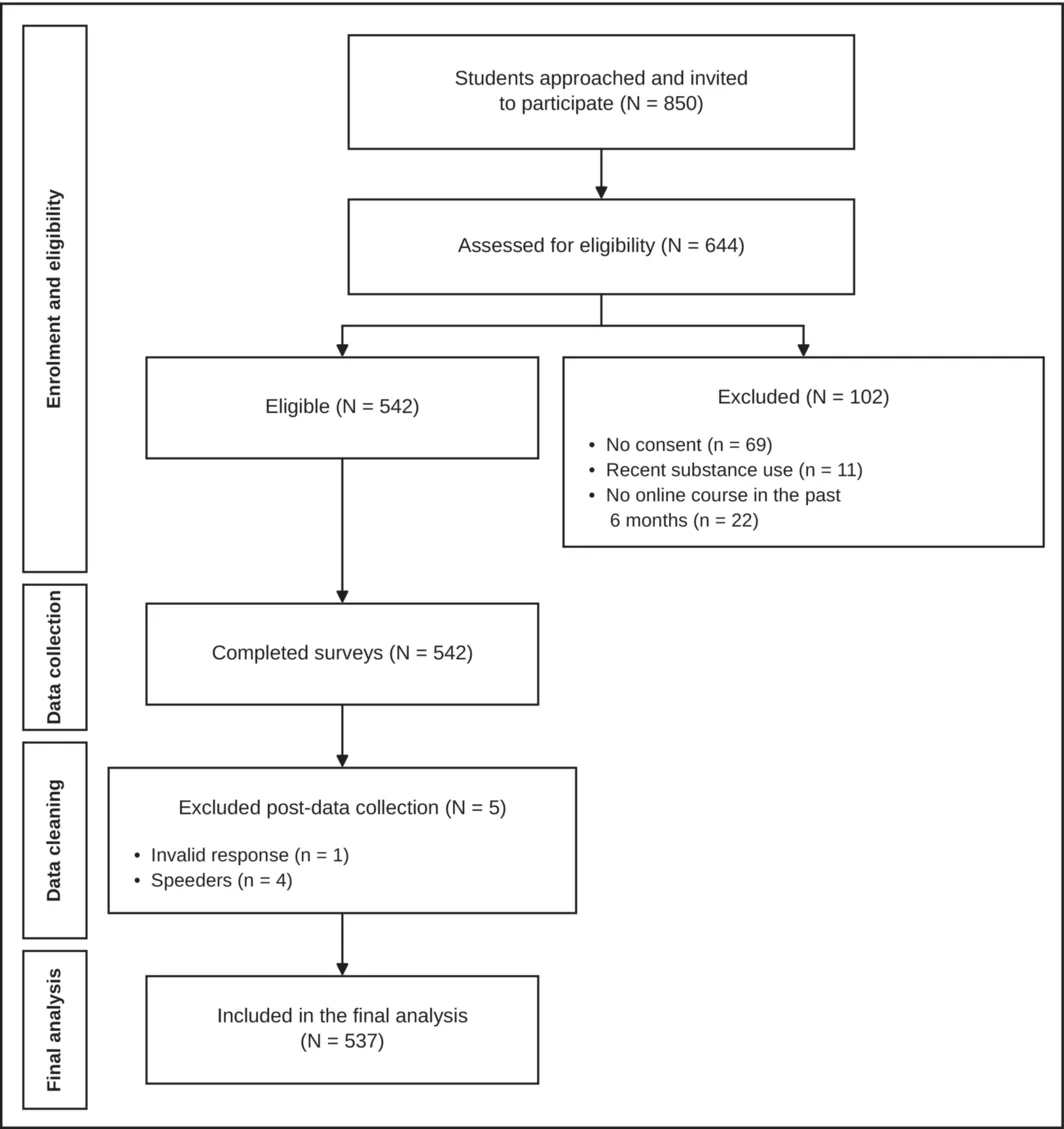
Flow diagram for participant recruitment and selection.

A total of 850 undergraduate nursing students were invited to participate through institutional learning platforms and student communication channels. Of these, 644 students accessed the questionnaire. After applying the eligibility criteria, 542 participants were eligible. Following data cleaning (five questionnaires were excluded, one invalid response and four speeders), 537 questionnaires were retained for the final analysis, yielding an overall response rate of 63.2% (Figure 1). As the online questionnaire required participants to complete all items before submission, no missing data were identified among the retained questionnaires.

### 2.2 Instruments

#### 2.2.1 Socio‐Demographic

The first part of the research gathered students' socio‐demographic information (age, sex, academic year, GPA, type of digital device, number of hours per day they use technology to study and internet quality).

#### 2.2.2 Zoom Exhaustion and Fatigue (ZEF) Scale

The second part of the survey included the ZEF scale to measure DF. The ZEF is a 15‐item questionnaire that comprises five subscales to measure fatigue: (1) general (items 1–3), (2) social (items 4–6), (3) emotional (items 7–9), (4) visual (items 10–12) and (5) motivational (items 13–15). The items are scored on a five‐point Likert scale, from 1 (not at all) to 5 (Extremely). Scores were between 15 and 75, and higher values indicate more DF. The ZEF scale demonstrated good internal consistency (Cronbach's *α* = 0.95) and is widely used in educational settings (Fauville et al. 2021).

#### 2.2.3 Cognitive Load Scale

The third section used the CL scale developed by Leppink et al. (2013). The CLS is a reliable tool for assessing types of CL (intrinsic CL, extrinsic CL and germane CL) by CLT during learning tasks. There are 10 items and each is rated on a scale of 1 (not at all the case) to 10 (completely the case). Items are grouped according to three components of CL: intrinsic load (3 items), extrinsic load (3 items) and germane load (4 items). The germane load items (7–10) were scored in reverse when the total score was calculated. The possible values range from 10 to 100 and higher scores indicate higher CL. The subscales seemed to have good internal consistency, with Cronbach's alpha values of 0.87 (intrinsic), 0.75 (extrinsic) and 0.77 (germane). This instrument has been used in several studies on online learning, medical education and instructional design (Klepsch et al. 2017; Leppink et al. 2013; Sweller et al. 2019).

### 2.3 Translation and Validation

The ZEF scale and CLS were translated into Arabic and validated for use in the Jordanian context, following cross‐cultural adaptation guidelines (Beaton et al. 2000). The process included the following steps: (1) forward translation: two bilingual, native Arabic‐speaking translators with different educational backgrounds independently carried out the translation from English to Arabic. One of them held a PhD in Education, while the other had a degree in Art. (2) synthesis: this phase involved resolving discrepancies in the forwarded translation, in the presence of a third expert who played the role of judge. (3) back translation: another two bilingual native English‐speaking translators who were blinded to the original instruments and their concepts back‐translated the instruments from Arabic to English and discrepancies were eliminated. (4) expert committee review: the purpose of this committee was to produce a pre‐final version by consolidating all versions of the instruments and achieving equivalence between the original and translated versions. This committee included experts in translation, education and validation.

After developing the pre‐final versions, five subject‐matter experts in education and psychology evaluated the instruments for clarity, relevance, and representativeness. The ZEF scale demonstrated strong content validity (I‐CVI ≥ 0.81 for all items; S‐CVI/Ave = 0.91), and the CLS showed similarly high values (I‐CVI ≥ 0.84 for all items; S‐CVI/Ave = 0.94).

Thirty‐two nursing students were recruited for pilot testing. The pilot testing (*n* = 32) was conducted as a preliminary validation phase to assess the clarity, reliability and feasibility of the instruments before the main data collection, rather than constituting a full psychometric validation study. The results showed high internal consistency for the ZEF (*α* = 0.89) and the CLS (*α* = 0.85), confirming their reliability and suitability for use among the Arabic‐speaking nursing student population.

### 2.4 Statistical Analysis

Data completeness was ensured at the point of collection, as all items within the online questionnaire were set as mandatory fields; therefore, no missing or incomplete responses were present in the dataset, and no missing data handling procedures (e.g., imputation or case deletion) were required.

Data analysis was performed using SPSS version 27.0. Descriptive statistics summarised the continuous and categorical variables. Bivariate analyses, including Spearman's correlation, the Mann–Whitney *U* test and the Kruskal–Wallis *H* test, were conducted to assess the relationship between the participants' characteristics and the study variables. Spearman's correlation coefficient was used to measure the relationship between DF and CL. Correlation coefficients were interpreted according to Evans (1996) where 0.00–0.19 were considered very weak, 0.20–0.39 weak, 0.40–0.59 moderate, 0.60–0.79 strong and 0.80–1.00 very strong.

Multiple linear analyses were performed to evaluate whether participants' characteristics were related to DF or CL. Prior to model fitting, categorical variables containing sparse categories were reviewed and categories with a small number of observations were merged where conceptually appropriate to improve model stability and ensure reliable parameter estimation. In addition, categorical factors were transformed into dummy variables to enable comparisons between each category and the designated reference group within the regression model. Variables included in the regression models were selected based on their statistical significance in the bivariate analysis and their theoretical relevance as supported by previous literature. All selected variables were entered simultaneously using the Enter method.

Before conducting multiple regression analyses, the assumptions were checked for regression analysis including absence of multicollinearity, normality of residuals, linearity, independence of errors and homoscedasticity. As the data were not normally distributed, bootstrap analyses with 5000 resamples were performed to generate bias‐corrected 95% confidence intervals for the regression coefficients. A *p <* 0.05 was considered statistically significant in all statistical analyses.

## 3 Results

### 3.1 Participants' Characteristics

A final sample of 537 nursing students was involved in the study. The mean (SD) age was 20.82 (1.08) years, while 58.7% were female. Over half (56.8%) were students in private universities and approximately half of them were in their fourth year (50.7%). With respect to academic success, 34.5% of students reported a GPA range of 3.0–3.49. Approximately half of the students (56.1%) reported using digital devices for ≤ 3 h per day, with smartphones being the most frequently used digital device (50.8%). The percentage of students who reported good internet quality was 47.1% (see Table 1).

**TABLE 1:** Participants characteristics (*N* = 537).

Variable	(*n*)	(%)
Age M = 20.82, SD = 1.08		
Sex		
Male	222	41.3
Female	315	58.7
Type of university		
Governmental	232	43.2
Private	305	56.8
Academic year		
First & Second year	141	26.3
Third year	124	23.1
Fourth year	272	50.7
GPA		
2.0–2.49 or 65%–69%	65	12.1
2.5–2.99 or 70%–74%	128	23.8
3.0–3.49 or 75%–84%	185	34.5
3.5–4.0 or 85%–100%	159	29.6
Daily device use		
≤ 3 h	301	56.1
4–6 h	138	25.7
> 6 h	98	18.2
Device used		
Smartphone	273	50.8
Tablet, PC, or laptop	264	49.2
Internet quality		
Weak	92	17.1
Good	253	47.1
Very Good	97	18.1
Excellent	95	17.7

*Note: n*, frequency; %, percentage.

### 3.2 Descriptive Statistics of DF and CL

Table 2 presents the observed median (IQR) and minimum and maximum scores. Total DF scores ranged from 31 to 75 (Mdn = 61.00, IQR = 55.0–67.0), with the scores clustering toward the upper portion of the possible range, suggesting that participants generally reported moderate to high levels of DF. For total CL, scores ranged from 32 to 75 (Mdn = 56.00, IQR = 51.0–61.0). Among the CL subscales, intrinsic CL ranged from 6 to 30 (Mdn = 16.00, IQR 14.0–22.0) and germane CL ranged from 4 to 28 (Mdn = 12.00, IQR = 10.0–14.0).

**TABLE 2:** Descriptive statistics of study variables (*N* = 537).

Variable	Minimum	Maximum	Median	IQR (25th–75th percentile)
Total DF	31	75	61.00	55.0–67.0
Total CL	32	75	56.00	51.0–61.0
Intrinsic CL	6	30	16.00	14.0–22.0
Extrinsic CL	7	30	30.00	26.0–30.0
Germane CL	4	28	12.00	10.0–14.0

*Note:* The observed range of total cognitive load reflects participant level summit scores; therefore, the minimum and maximum values of the subscales do not necessarily occur within the same participants and should not be summed to derive the total score range.

Abbreviation: IQR, interquartile range.

Extrinsic CL showed a markedly different pattern from the other subscales. Although scores ranged from 7 to 30, both the median (30.00) and the 75th percentile (30.0) coincided with the maximum possible values on the scale. This indicates that at least half of the sample scored at the ceiling of the extrinsic CL measure. This pronounced ceiling effect provides clear empirical justification for the bootstrapping approach adopted in the subsequent regression analysis, as it confirms that the extrinsic CL variable was substantially non‐normally distributed and heavily skewed toward its upper bound.

### 3.3 Association Between DF, Total CL, and CL Subscales

Spearman's correlation analysis revealed a significant positive correlation between total DF and total CL (*r*_*s*_ = 0.405, *p* < 0.001), indicating that higher levels of DF were associated with greater overall CL. Regarding the CL subcomponents, there was a positive association between intrinsic CL and DF (*r*_*s*_ = 0.224, *p* < 0.001), a positive association with extrinsic CL (*r*_*s*_ = 0.308, *p* < 0.001) and a negative association with germane CL (*r*_*s*_ = −0.118, *p* < 0.01). The results imply that extrinsic instructional variables were most strongly correlated with DF and were negatively correlated with students' meaningful learning engagement.

### 3.4 Association Between Participants' Characteristics and Study Variables

Table 3 shows the associations between participants' characteristics and extrinsic CL. The majority of the groups across all variables had a median extrinsic CL score of 30.00. Significant associations were found only for academic year (H = 14.948, *p* = 0.001). No significant associations were observed for age, sex, type of university, GPA, daily device use, device used, or Internet quality (*p* > 0.05).

**TABLE 3:** Association between participants' characteristics and extrinsic CL.

Variables	rho/Median	*p*
Age^a^	0.054	0.064
Sex^b^		0.364
Male	30.00	
Female	30.00	
Type of university^b^		0.820
Governmental	30.00	
Private	30.00	
Academic year^c^		0.001
First & Second year	30.00	
Third year	29.00	
Fourth year	30.00	
GPA^c^		0.271
2.0–2.49 or 65%–69%	30.00	
2.5–2.99 or 70%–74%	30.00	
3.0–3.49 or 75%–84%	30.00	
3.5–4.0 or 85%–100%	30.00	
Daily device use^c^		0.115
≤ 3 h	30.00	
4–6 h	30.00	
> 6 h	30.00	
Device used^b^		0.166
Smartphone	30.00	
Tablet, PC, or laptop	30.00	
Internet quality^c^		0.928
Weak	30.00	
Good	30.00	
Very Good	30.00	
Excellent	30.00	

^a^ Spearman correlation.

^b^ Mann–Whitney *U* test.

^c^ Kruskal–Wallis *H* test; rho, Spearman correlation.

### 3.5 Factors Association With Extrinsic CL

A multiple linear regression analysis with bootstrap estimation was conducted to identify factors independently associated with extrinsic CL. The overall regression model was statistically significant, *F*(15, 521) = 15.35, *p* < 0.001, explaining 30.7% of the variance in extrinsic CL (*R* ^2^ = 0.307; adjusted *R* ^2^ = 0.287) (see Table 4).

**TABLE 4:** Multiple linear regression model of factors associated with extrinsic CL.

Variables	*B*	SE	*p*	95% BCa CI	
				Lower	Upper
Constant	3.580	6.508	0.581	−9.444	16.370
Age	0.255	0.284	0.368	−0.297	0.813
Sex					
Male	−0.390	0.500	0.440	−1.352	0.603
Female (reference)					
Type of university					
Governmental	−2.686	0.845	0.002	−4.385	−1.021
Private (reference)					
Academic year					
First & Second year	−1.278	0.983	0.188	−3.221	0.687
Third year	−2.925	0.909	0.001	−4.688	−1.127
Fourth year (reference)					
GPA					
2.0–2.49 or 65%–69%	0.620	0.683	0.373	−0.712	1.958
2.5–2.99 or 70%–74%	0.727	0.586	0.222	−0.403	1.885
3.0–3.49 or 75%–84%	−0.564	0.562	0.317	−1.679	0.534
3.5–4.0 or 85%–100% (reference)					
Daily device use					
≤ 3 h	0.010	0.779	0.990	−1.566	1.513
4–6 h	−1.605	0.790	0.039	−3.167	−0.089
> 6 h (reference)					
Device used					
Smartphone	−1.173	0.867	0.175	−2.887	0.558
Tablet, PC, or laptop (reference)					
Internet quality					
Weak	0.431	0.801	0.587	−1.187	1.962
Good	−0.180	0.733	0.812	−1.567	1.282
Very Good	−0.467	0.781	0.545	−2.013	1.087
Excellent (reference)					
Total DF	0.342	0.033	< 0.001	0.275	0.404

Abbreviations: 95% BCa CI, Bias‐Corrected and accelerated Bootstrap Confidence Interval; B, Unstandardised Coefficient; p, statistical significance at < 0.05; SE, Standard Error.

After controlling for participants' demographic and other characteristics, DF was strongly associated with extrinsic CL (B = 0.342, *p* < 0.001, 95% BCa CI [0.275, 0.404]). Compared with fourth‐year students, third‐year students demonstrated significantly lower extrinsic CL (B = −2.925, *p* = 0.001, 95% BCa CI [−4.688, −1.127]). Students attending governmental universities reported significantly lower extrinsic CL than those attending private universities (B = −2.686, *p* = 0.002, 95% BCa CI [−4.385, −1.021]). Regarding daily digital device use, students who use digital devices for 4–6 h per day exhibited significantly lower extrinsic CL than those using them for more than 6 h per day (*B* = −1.605, *p* = 0.039, 95% BCa CI [−3.167, −0.089]).

## 4 Discussion

The present study examined DF, CL and factors associated with these constructs among nursing students in Jordanian universities. Overall, the results show that the students experienced relatively high DF and CL levels, with extrinsic CL emerging as the dominant cognitive dimension and showing the strongest association with DF. These results together indicate that students' online learning experiences may reflect not only the extent of digital engagement and the time spent in digital learning but also the cognitive demands imposed by instructional design and the learning environment (Basch et al. 2025; Bennett et al. 2021; Peper et al. 2021).

An important finding of the present study was the pronounced ceiling effect observed for extrinsic CL, with at least half of the participants scoring at the upper limit of the scale. According to CLT, excessive extrinsic CL reflects inefficiencies in instructional design rather than the inherent complexity of learning material and may reduce the cognitive resources available for meaningful learning (Sweller et al. 2019; Mayer 2021).

In the online nursing environment, this may stem from fragmented platforms, frequent task switching, unclear navigation and repetitive screen‐based interaction, which impose unnecessary cognitive processing beyond what the learning content itself (Hung et al. 2024; Le Cunff et al. 2024; Paas and Sweller 2014). By contrast, germane CL–the cognitive effort directed toward schema building and meaningful learning–was inversely associated with DF, suggesting that as unnecessary extrinsic demands may coexist fewer cognitive resources remain available for deep, meaningful engagement with the material (Haanes et al. 2024; Huang and Fang 2023). Taken together, these findings point to instructional design, rather than digital engagement in itself, as the primary modifiable source of cognitive burden, highlighting the importance of reducing extrinsic task demands to promote learning and engagement (Mayer 2021; Sepp et al. 2019).

The positive association observed between DF and total CL is consistent with CLT, which proposes that excessive cognitive demands may exceed working memory capacity and reduce learning efficiency (Sweller et al. 2019). Prolonged digital engagement may intensify mental effort and information‐processing demands (Bailenson 2021a; Xue et al. 2024). Given the cross‐sectional design, these findings should be interpreted as associations rather than causal relationships (Bailenson 2021b; Sweller et al. 2019). Notably, the association between DF and total CL remained significant after adjustment for demographic and academic characteristics, suggesting that an association remained independent of these measured variables.

The finding that students in governmental universities experienced lower extrinsic CL than those in private universities has not been widely examined in previous research, as a few studies have directly compared institutional differences in CL. Notably, this association was not significant in the bivariate analysis (Table 3) but emerged as significant only after adjusting for other variables, particularly DF, in the multiple regression model. This pattern may reflect a statistical adjustment effect, whereby the relationship between university type and extrinsic CL was masked by shared variance with other predictors in the unadjusted analysis. This finding requires confirmation in future studies. It may reflect differences in instructional organization, curriculum delivery, or digital learning implementation across institutions.

The present study found that third‐year students reported lower extrinsic CL than fourth‐year students. Although direct evidence comparing CL across academic years remains limited, this finding may be interpreted in the context of nursing education, where students experience increasing academic and clinical complexity in later years. Previous qualitative evidence has highlighted that nursing students often experience fragmented integration across academic and clinical settings, particularly in higher academic year, due to frequent transitions between learning environments, clinical placements and peer groups, which may increase overall learning demands and reduce cognitive efficiency (Sweetman et al. 2022).

Compared with the students who used digital devices for more than 6 h per day, those using digital devices for 4–6 h per day reported significantly lower extrinsic CL. This finding is consistent with previous studies reporting that prolonged screen exposure is associated with increased mental effort, attentional demands, DF and reduced cognitive efficiency during online learning (Bailenson 2021b; Haanes et al. 2024; Surbakti et al. 2024).

On the other hand, the perceived quality of internet was not related to extrinsic CL here. While the connectivity issue has long been cited as a barrier for digital education, recent research has indicated that CL may be more strongly influenced by curriculum design and information organization than technical access alone (Skulmowski and Xu 2022).

Collectively, these findings suggest that instructional and pedagogical characteristics may contribute more substantially to students' CL than demographic or contextual characteristics.

## 5 Implications of the Study

This work has important implications for nursing education practice and curriculum design. Rather than focusing solely on digital technology use, educators should also look at improving the design of their teaching materials (i.e., the content they present) to reduce the CL as they work in an online learning setting. Strategies based on CLT have been used to better navigate; to break the learning down into smaller pieces, to minimise task switching; and to schedule time to process and recover from learning.

At the faculty level, to make online learning more cognitively based and student‐centered, training and capacity‐building programs should be implemented to enhance the teachers' digital pedagogy, teaching skills and instructional design. This can help enhance student learning efficiency, decrease perceived DF and retain students' interest.

## 6 Limitations of the Study

There are a few limitations of the study. The ceiling effect in extrinsic CL, where a large proportion of the participants scored near the upper limit of the scale, may have reduced the variability of responses and hence made the analysis process more sensitive to this outcome even though bootstrapping procedures were adopted. Second, while the regression model explained 30.7% of the variance of extrinsic CL (*R* ^2^ = 0.307), a great deal of the variance remained unexplained, suggesting additional factors not measured in the present study may contribute to students' cognitive experiences in online learning environments.

Third, the cross‐sectional design limits the ability to establish causal relationships between DF and CL. Fourth, several potential sources of bias warrant consideration. Convenience sampling may have introduced selection bias, as participants were recruited from students who were reachable through institutional learning platforms and communication channels during the data collection period. Consequently, students who were less engaged with institutional online communication systems may have been underrepresented, which may limit the generalizability of the findings to the broader population of nursing students in Jordan. In addition, the online, self‐administered survey approach may have introduced volunteer or self‐selection bias. Students who chose to participate may have differed from non‐respondents in characteristics related to digital learning experiences, digital fatigue, or cognitive load. For example, students with particularly positive or negative experiences of online learning may have been more likely to participate, potentially influencing the observed levels and associations of the study variables. Because information regarding non‐respondents was unavailable, the direction and magnitude of this potential bias cannot be determined.

Fifth, the reliance on self‐reported measures and subjective assessment of internet quality may have introduced reporting bias and may not fully reflect actual connectivity characteristics. Sixth, although pilot testing of these studies indicated that the results were valid and reliable, further psychometric measurements in larger and more diverse samples are required. Finally, unmeasured factors such as psychological stress, sleep quality and academic workload may have influenced the outcomes.

These limitations should be considered when interpreting the findings. Future research using probability‐based sampling methods, longitudinal designs, objective measures of digital learning conditions and comparisons between respondents and non‐respondents may help clarify the influence of these factors on students' DF and CL.

## 7 Conclusion

This study indicates that nursing students reported relatively elevated levels of DF and CL, with extrinsic CL emerging as the most prominent cognitive dimension associated with these experiences. The findings suggest that cognitive burden in online learning may be influenced by instructional design and learning conditions.

Improving instructional design and reducing unnecessary cognitive demands may enhance learning efficiency and reduce fatigue in online nursing education. Future interventions should focus on developing cognitively sustainable and pedagogically optimised digital learning environments.

## Author Contributions

**Ala'a Ayman Al‐yyan:** data curation. **Qusai Abdulrahman AbuQamar:** writing – original draft, writing – review and editing. **Randa Khirfan:** writing – review and editing. **Ahmad I. Miqdadi:** conceptualization, methodology, formal analysis, data curation. **Nawras Fashafsheh:** writing – review and editing. **Yousef Abu‐Wardeh:** formal analysis, data curation. **Shaban Sinnokrot:** conceptualization, writing – original draft, formal analysis, methodology.

## Funding

The authors received no specific funding for this study from any funding agency in the public, commercial, or not‐for‐profit sectors.

## Ethics Statement

Undergraduate nursing students from five nursing colleges (two governmental and three private) in Jordan were invited to participate, including the University of Jordan, Yarmouk University, Al‐Zaytoonah University of Jordan, Zarqa University, and Applied Science Private University. All procedures performed in the study involving human participants were in accordance with the ethical standards of the institutional and/or national research committee and with the 1964 Helsinki declaration and its later amendments or comparable ethical standards. The study was ethically approved by the Research Ethics Committee, Faculty of Nursing, Zarqa University (74/2024–2025), which had oversight of ethical approval for all participating institutions; no separate ethical approval was required from them. To facilitate data collection, an official letter of facilitation was obtained from Zarqa University and submitted to the Ministry of Higher Education and Scientific Research, which subsequently communicated with the participating universities to facilitate access to the institutions and data collection. The necessary administrative permissions were obtained from the relevant authorities at each participating institution prior to data collection.

## Consent

Permission to use and translate the study questionnaires was obtained from the respective instrument developers.

## Conflicts of Interest

The authors declare no conflicts of interest.

## Supporting information


**Supporting Information S1:** STROBE Statement—Checklist of items that should be included in reports of cross‐sectional studies, indicating where each item is reported in the manuscript.

## Data Availability

The data that support the findings of this study are available on request from the corresponding author. The data are not publicly available due to privacy or ethical restrictions.
