# Supplementary material for: Digital Fatigue and Cognitive Load in E‐Learning: Evidence From Nursing Students in Jordan
Source: Nurs Open. 2026 Sep 2;13(9):e70795. doi: 10.1002/nop2.70795 (PMC13537813; doi:10.1002/nop2.70795)
Supplement: Supplementary file 1 [file NOP2-13-e70795-s001.docx]

**Supplementary Material S1.** STROBE Statement — Checklist of items that should be included in reports of cross-sectional studies, indicating where each item is reported in the manuscript.

***Manuscript: Digital Fatigue and Cognitive Load in E-Learning: Evidence from Nursing Students in Jordan***

| **Item** | **STROBE Recommendation** | **Location in Manuscript** | **Page No.** |
| --- | --- | --- | --- |
| **Title and abstract** | | | |
| **1a** | Indicate the study's design with a commonly used term in the title or the abstract | Design is named in the structured abstract's "Design" field ("A cross-sectional study"); not stated in the title itself. | *p. 1* |
| **1b** | Provide an informative and balanced summary of what was done and what was found | Structured abstract (Aim, Design, Methods, Results, Conclusion, Implications, Impact). | *pp. 1–2* |
| **Introduction** | | | |
| **2** | Explain the scientific background and rationale for the investigation being reported | Introduction (digital fatigue and cognitive load in online nursing education; gap in literature on multidimensional CL and DF in nursing students in resource-limited settings). | *pp. 3–5* |
| **3** | State specific objectives, including any prespecified hypotheses | Final paragraph of Introduction: "guided by CLT, this study aimed to assess DF and multidimensional CL, and to examine their associated factors..." | *p. 5* |
| **Methods** | | | |
| **4** | Present key elements of study design early in the paper | Section 2.1, first sentence: "This cross-sectional study presented an internet-based questionnaire..." | *pp. 5–6* |
| **5** | Describe the setting, locations, and relevant dates, including periods of recruitment, exposure, follow-up, and data collection | Section 2.1: governmental/private nursing colleges in Jordan; data collected March–April 2025. | *p. 6* |
| **6a** | Give the eligibility criteria, and the sources and methods of selection of participants | Section 2.1: inclusion/exclusion criteria; convenience sampling of undergraduate nursing students via institutional platforms. | *p. 6* |
| **7** | Clearly define all outcomes, exposures, predictors, potential confounders, and effect modifiers | Section 2.2 (socio-demographics, ZEF subscales, CLS subscales) and Section 2.4 (variables entered in regression selected by bivariate significance/theoretical relevance). | *pp. 7–8, 10* |
| **8** | For each variable of interest, give sources of data and details of methods of assessment (measurement) | Section 2.2.1–2.2.3: ZEF scale (Fauville et al., 2021) and Leppink CLS (Leppink et al., 2013) described with subscales, scoring ranges, and reliability (Cronbach's α). | *pp. 7–8* |
| **9** | Describe any efforts to address potential sources of bias | Section 2.3 (translation/validation, expert review, pilot testing). Broader bias discussion (selection/self-selection) appears in Section 6 (Limitations) rather than Methods. | *pp. 8–9 (Methods); pp. 19–20 (Limitations)* |
| **10** | Explain how the study size was arrived at | Section 2.1: G*Power 3.1 calculation (α = .05, f² = .15, power = .90, 15 predictors) → minimum required n = 171. | *p. 7* |
| **11** | Explain how quantitative variables were handled in the analyses; describe which groupings were chosen and why | Section 2.2 (scale scoring/ranges); Section 2.4 (sparse categorical levels merged for model stability; categorical variables dummy-coded against reference groups). | *pp. 7–8, 10* |
| **12a** | Describe all statistical methods, including those used to control for confounding | Section 2.4: bivariate tests (Spearman, Mann–Whitney U, Kruskal–Wallis) followed by multiple linear regression with all selected variables entered simultaneously (Enter method). | *pp. 9–10* |
| **12b** | Describe any methods used to examine subgroups and interactions | Not explicitly described (no interaction terms reported). | *Not addressed* |
| **12c** | Explain how missing data were addressed | Section 2.4, first line: all questionnaire items mandatory, so no missing/incomplete responses; no imputation/case deletion required. | *p. 9* |
| **12d** | If applicable, describe analytical methods taking account of sampling strategy | Not applicable (non-probability/convenience sampling; no weighting used) — could be stated explicitly as not applicable. | *Not addressed* |
| **12e** | Describe any sensitivity analyses | Section 2.4 / 3.5: bootstrap analysis with 5,000 resamples for bias-corrected 95% CIs, used because regression assumptions (normality) were not met. | *pp. 10, 14* |
| **Results** | | | |
| **13a** | Report numbers of individuals at each stage of the study (e.g., numbers potentially eligible, examined for eligibility, confirmed eligible, included in the study, completing follow-up, and analyzed) | Section 2.1 / Figure 1: 850 invited → 537 accessed, eligible, and completed (63.2% response rate); all 537 included in analysis. | *p. 6* |
| **13b** | Give reasons for non-participation at each stage | Section 2.1: single point of attrition described as non-response between invitation and completion (reasons for non-response itself not detailed). | *p. 6 — partially addressed* |
| **13c** | Consider use of a flow diagram | Figure 1 (participant flow diagram) is referenced twice in Section 2.1. The diagram itself is maintained as a separate file per journal submission requirements, not embedded in the manuscript file. | *p. 6 (in-text reference); Figure 1 submitted as separate file* |
| **14a** | Give characteristics of study participants (e.g., demographic, clinical, social) and information on exposures and potential confounders | Section 3.1 and Table 1 (age, sex, university type, academic year, GPA, device use, device type, internet quality). | *pp. 10–11* |
| **14b** | Indicate the number of participants with missing data for each variable of interest | Section 2.4/3.1: no missing data (all fields mandatory) — explicitly stated. | *pp. 9–10* |
| **14c** | Cross-sectional study: summarize follow-up time | Not applicable — no follow-up period (single time-point cross-sectional design). | *N/A* |
| **15** | Cross-sectional study: report numbers of outcome events or summary measures | Section 3.2 and Table 2 (median, IQR, min/max for total DF, total CL, and CL subscales). | *pp. 11–12* |
| **16a** | Give unadjusted estimates and, if applicable, confounder-adjusted estimates and their precision (95% CI); make clear which confounders were adjusted for and why they were included | Section 3.4/Table 3 (unadjusted/bivariate associations) and Section 3.5/Table 4 (adjusted regression estimates with bootstrapped 95% BCa CIs). | *pp. 13–15* |
| **16b** | Report category boundaries when continuous variables were categorized | Table 1/Table 3: category boundaries given for academic year, GPA, daily device use, internet quality. | *pp. 10–11, 13–14* |
| **16c** | If relevant, translate estimates of relative risk into absolute risk for a meaningful time period | Not applicable — outcome is continuous (extrinsic CL score), not a risk/event outcome. | *N/A* |
| **17** | Report other analyses done (e.g., analyses of subgroups and interactions, sensitivity analyses) | Section 3.5/Table 4: bootstrap sensitivity analysis (5,000 resamples) reported as the primary inferential approach. | *pp. 14–15* |
| **Discussion** | | | |
| **18** | Summarize key results with reference to study objectives | Discussion, opening paragraph: DF and CL levels, extrinsic CL as dominant/most associated dimension. | *pp. 15–16* |
| **19** | Discuss limitations of the study, taking into account sources of potential bias or imprecision; discuss both direction and magnitude of any potential bias | Section 6 (Limitations): ceiling effect, unexplained variance, cross-sectional design, convenience/self-selection bias, self-report/reporting bias, pilot-only psychometric testing, unmeasured confounders. Direction/magnitude of self-selection bias explicitly noted as indeterminable. | *pp. 19–20* |
| **20** | Give a cautious overall interpretation considering objectives, limitations, multiplicity of analyses, results from similar studies, and other relevant evidence | Discussion, throughout (e.g., explicit caveat that cross-sectional findings are associations, not causal relationships). | *pp. 16–18* |
| **21** | Discuss the generalizability (external validity) of the study results | Section 6: convenience sampling and institutional-channel recruitment flagged as limiting generalizability to the broader Jordanian nursing student population. | *pp. 19–20* |
| **Other information** | | | |
| **22** | Give the source of funding and the role of the funders for the present study and, if applicable, for the original study on which the present article is based | Funding section (following the Conclusion and before the References): "This research received no specific grant from any funding agency in the public, commercial, or not-for-profit sectors." | *p. 21* |
